# Oxidative-antioxidative status and hepatic and renal factors following melatonin administration in castrated and intact dogs

**DOI:** 10.1186/s12610-019-0094-6

**Published:** 2019-11-15

**Authors:** Asghar Mogheiseh, Farzaneh Koohi, Saeed Nazifi, Aidin Shojaee Tabrizi, Pegah Taheri, Sina Salavati

**Affiliations:** Department of Clinical Sciences, School of Veterinary Medicine, Shiraz University, P.O.Box 71441-69155, Shiraz, Iran

**Keywords:** Castration, Melatonin, Oxidative stress, Antioxidant, Castration, Mélatonine, Stress oxydant, Anti-oxydant

## Abstract

**Backgrounds:**

Melatonin has significant antioxidant and hepatoprotective effects in normal and oxidative stress conditions. The aim of the present study was to assess the effects of melatonin on antioxidant, hepatic, and renal factors in intact and castrated dogs. Twenty male mixed-breed adult dogs were aligned in an experimental randomized and controlled trial. The dogs were randomly divided into four equal groups: melatonin, castrated, castrated and melatonin, and control. They were treated with melatonin (0.3 mg/Kg, once daily, orally) immediately after the castration for 1 month and their blood samples were collected weekly from 2 days after treatment with melatonin.

**Results:**

Treating castrated dogs with melatonin increased the level of glutathione peroxidase, superoxide dismutase, and catalase compared with that of the control and castrated groups. The malondialdehyde level increased significantly following castration. Melatonin treatment decreased malondialdehyde concentration in the castrated dogs. Castration increased the level of alkaline phosphatase, aspartate aminotransferase, and alanine aminotransferase significantly in comparison with that of the control group. Treating the castrated dogs with melatonin decreased significantly liver enzymes compared with those of the castrated dogs. Blood urea nitrogen and creatinine levels increased in the castrated dogs in comparison with that of the control group.

**Conclusions:**

The administration of melatonin in castrated dogs increased antioxidant activity and decreased oxidation products, compared with those of the castrated and untreated dogs, without adverse effects on liver enzymes and kidney function.

## Résumé

**Contexte:**

La mélatonine possède des effets anti-oxydants et hépato-protecteurs dans les conditions normales et dans les situations de stress oxydant. L’objectif de la présente étude est d’évaluer les effets de la mélatonine sur des facteurs anti-oxydants, hépatiques et rénaux chez des chiens intacts et castrés. Vingt chiens mâles adultes de race croisée ont été utilisés dans un essai expérimental randomisé avec groupe témoin. Les chiens ont été attribués au hasard à quatre groupes de même effectif : mélatonine, castré, castré et mélatonine, et témoin. Ils ont été traité par la mélatonine (0.3 mg/kg, une fois par jour, par voie orale) immédiatement après la castration, pendant un mois ; des échantillons sanguins ont été collectés une fois par semaine à partir du deuxième jour de traitement par la mélatonine.

**Résultats:**

Le traitment par mélatonine chez les chiens castrés augmente les niveaux de glutathion peroxydase, de superoxyde dismutase, et de catalase par comparaison avec les niveaux chez les groupes témoin et castré. Le niveau de malondialdéhyde augmente de façon significative après la castration. Le traitement par mélatonine diminue la concentration de malondialdéhyde chez les chiens castrés. La castration augmente significativement les niveaux de phosphatase alcaline, d’aspartate aminotransférase, et d’alanine aminotransférase par comparaison à ceux du groupe témoin. Le traitement des chiens castrés par la mélatonine diminue de façon significative les enzymes hépatiques par comparaison à ceux des chiens castrés. Les taux sanguins d’azote uréique et de créatinine augmentent chez les chiens castrés comparé à ceux des chiens témoins.

**Conclusions:**

L’administration de mélatonine à des chiens castrés augmente l’activité anti-oxydante, et diminue les produits d’oxydation, par comparaison avec les chiens du groupe castré et non traité, sans effets indésirables sur les enzymes hépatiques et sur la fonction rénale.

## Background

Oxidative stress could disturb many physiological processes, including cell damages and the promotion of apoptosis [1]. The antioxidant effects of melatonin are significant and can influence antioxidant enzyme activity and the cellular mRNA levels for these enzymes either under physiological or elevated oxidative stress conditions [2]. In experimental studies that acetylsalicylic acid was used to induce oxidative stress [3], it was reported that aluminum chloride [4], lead [5], chlorpyrifos-ethyl [6], and melatonin metabolites could protect tissues against the oxidative damages because the toxic materials, metabolic processes, and melatonin were able to balance the antioxidant components to decrease the side effects of oxidation [7]. Melatonin and its metabolites are multifunctional indolamine. The beneficial effects of melatonin on free radicals and peroxynitrite-induced cellular toxicity have been revealed in numerous experimental and clinical studies [8, 9]. Melatonin was more effective than the classical antioxidants (e.g., vitamins E and C) in protecting the body against oxidative/nitrosative stress [10].

Hepatocellular enzymes, such as alanine aminotransferase (ALT) and aspartate aminotransferase (AST), are released following liver damage. Alkaline phosphatase (ALP) is markedly elevated in response to cholestasis. The hepatoprotective effects of melatonin, such as acetylsalicylic acid-induced liver damage in rats [11], rats fed with a high-fat ratio [12], hepatic necrosis in streptozotocin-induced diabetic rats [13], and tramadol-induced hepatotoxicity in albino rats [14], have been confirmed in experimental studies. Oxidative stress and inflammation induced during aging in the liver have been more marked in castrated than in the intact female rats. The administration of melatonin reduced both adverse conditions [15]. This protective effect of melatonin has been reported against oxidative damage induced by aluminum in rats’ kidneies [16], acetylsalicylic acid-induced kidney and testis damage [17], decreased urinary excretion of n-acetyl-bd-glucosaminidase, and albumin and renal oxidative markers in diabetic rats [18].

Surgical procedures, such as laparoscopic and open ovariectomy, have been demonstrated to increase plasma total oxidant status and oxidative stress index and decrease the total antioxidant status in dogs [19]. The more oxidative stress, the more severe tissue damages would be during surgery and that may cause poor outcomes in patients [20]. Thus, controlling oxidative stress after surgeries could decrease the side effects following the physiological stress response and that includes the activation of inflammatory, endocrine, metabolic, and immunological mediators [21].

Surgical procedures induce oxidative stress and inflammatory processes that could lead to short and long side effects [21]. Castration is one of the most common procedures for population control and treatment of testosterone-related diseases in dogs. Postoperative care (1 month) should be performed to reduce the side effects [22]. Thus, we proposed that the administration of melatonin will ameliorate the potential oxidative stress and inflammation induced by castration, compared to the control and castrated dogs.

## Materials and methods

### Statement of animal rights

All animal experiments were approved by the State Committee on Animal Ethics, Shiraz University, Shiraz, Iran (IACUC no: 4687/63). The recommendations of European Council Directive (2010/63/EU) of September 22, 2010, regarding the standards in the protection of animals used for experimental purposes, were also followed.

### Animals and experimental design

**Fig. 1:**
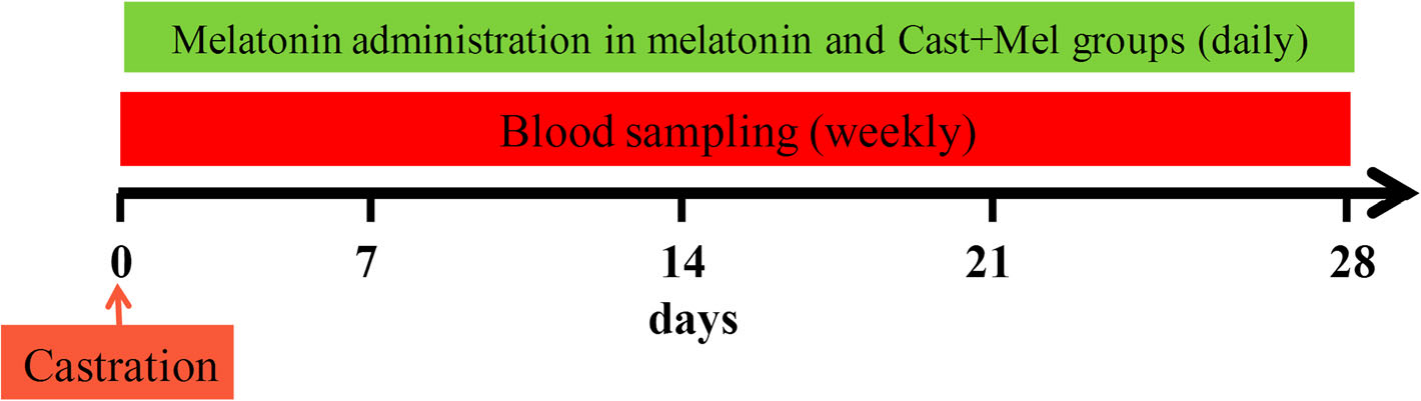
Schematic drawing of the study design. Twenty intact male adult dogs were aligned into four equal groups (*n* = 5): melatonin, castrated, control, and castrated with melatonin treatment (Cast+Mel). Ten dogs were castrated (castrated and Cast+Mel groups) and ten dogs were treated with melatonin (melatonin and Cast+Mel groups)

Twenty intact male-mixed breed stray dogs, aged 2.5 (1–3) years old, with a mean body weight of 20 (18–24 kg) were selected for this study. During 2 weeks of preparation, all animals were dewormed (Praziquantel; 50 mg/kg; Mebendazole; 100 mg/kg) and received 12 h of light by a digital light timer and 300 g/dog/day of commercial dog food daily (NUTRI Dry Dog Food; Behintash Co. Iran). The dogs were randomly divided into four equal groups (*n* = 5). The melatonin group received oral melatonin capsule (3 mg, L’ORGANIQUE, Canada) daily for 1 month. The castrated group was castrated using the orchiectomy method and did not receive melatonin. The Cast+Mel group included dogs which were castrated by the orchiectomy method and received melatonin capsule daily for 1 month. The control group received neither melatonin nor castration (Fig. 1). The dose for the oral melatonin administration was 0.3 mg/kg body weight, once a day, which began immediately after recovery from anesthesia and continued for 1 month [23]. Post-surgical and full recovery time after castration was considered for about 1 month. Ten milliliters of blood was taken from each dog’s jugular vein into simple glass tubes and ethylenediaminetetraacetic acid (EDTA) vacutainer tubes weekly; this was done from two days after the start of melatonin administration and continued for 1 month (first sampling = day 0 of study). Blood samples were sent to a laboratory with ice bags because of the warm weather. Sera were extracted for biochemical assays using 3000 × rpm centrifugation for 10 min within 2 hours after sampling and kept in a − 20 °C. Also, for assaying the glutathione peroxidase (GPX), superoxide dismutase (SOD), and catalase (CAT) activity, red blood cell (RBC) washing was performed three times by adding 3 ml of normal saline to 0.5 ml of blood samples from EDTA vacutainers and centrifuged for 10 min and 3000 × rpm. After the last washing and the removal of supernatant fluid, 2 ml of distilled water was added to RBC and all of them were kept at 4 °C for 15 min and stored at − 20 °C. All laboratory measurements were performed within 1 month after samplings.

### Castration method

All dogs were part of a population control program, so all of them were finally castrated and released in their living geographical area. Ten dogs were selected randomly for castration. Anesthesia premedication was done using acepromazine (0.05 mg/kg, IM, Alfasan, Woerden, Holland) and morphine (0.1 mg/kg, IM, Darou Pakhsh, Iran). The induction was performed using ketamine (5 mg/kg, IV, Alfasan, Woerden, Holland) and midazolam (0.2 mg/kg, IV, Darou Pakhsh, Iran). Isoflurane (1.2%, Nicholas Piramal Limited, London, UK) was used for maintenance. Then, orchiectomy was performed as an open procedure [24].

### Biochemical analysis

Biochemistry profiles, including blood urea nitrogen (BUN), Creatinine, Phosphorus, Total protein, ALP, ALT, AST, and Albumin, were obtained using standard methods and commercial kits (Pars Azmoon Kits, Iran) and biochemical auto analyzer (Alpha Classic AT^++^, Sanjesh, Iran*)*. All kits were validated for dog samples in the laboratory.

### Evaluation of oxidative stress indices

#### Antioxidant enzymes measurement

The SOD, GPX, and CAT activities were assessed by commercial kits (Zell Bio Company, Germany) in hemolyzed RBCs. The activities of the antioxidant enzymes were all stated as units per gram of hemoglobin. All kits were validated for dog samples in the laboratory.

#### Measurement of malondialdehyde (MDA)

MDA was measured using the ZellBio GmbH kit (Germany) based on its reaction with thiobarbituric acid in acidic conditions and high temperature, and the color complex was measured calorimetrically at 535 nm. The values were finally expressed as mmol/L. The mentioned kit was validated for dog samples in the laboratory.

#### Statistical analysis

Statistical analysis was performed by Graphpad Prism software, version 6. Normal distribution was confirmed by analyzing the data. The mean concentrations were compared using one-way repeated-measures analysis of variance (ANOVA) and Tukey’s multiple comparisons tests. The effects of time, group, and the interaction of time and group factors were analyzed by two-way repeated-measures ANOVA and Tukey’s multiple comparisons tests. All the data were presented as Mean ± SEM. *P* values less than 0.05 were considered as significant.

## Results

**Fig. 2:**
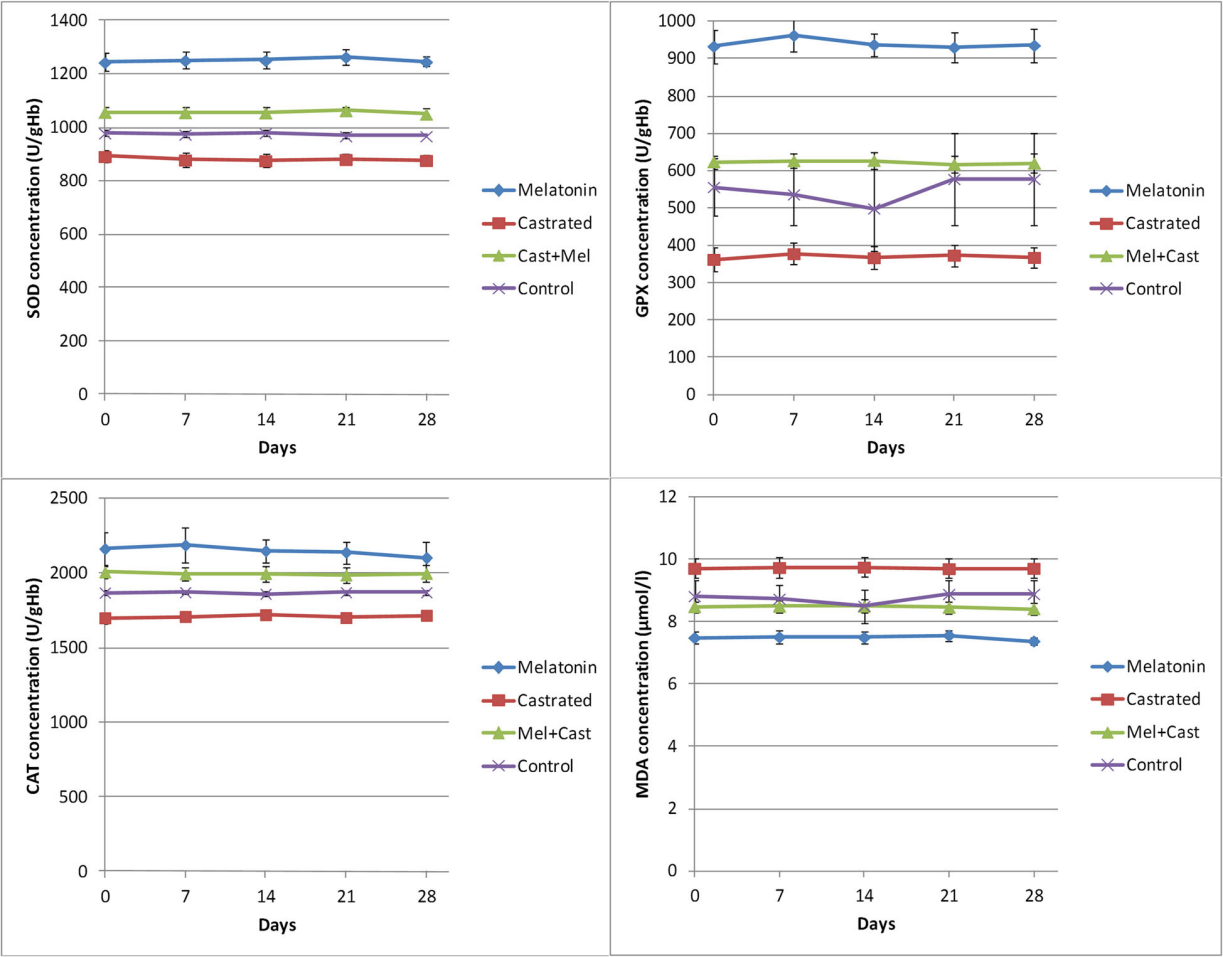
Changes in the antioxidant enzymes (SOD, GPX, and CAT) and malondialdehyde (MDA) concentrations (Mean ± SEM) in the control, melatonin, castrated, and castrated+melatonin groups (*n* = 5 in each group) during the one-month study. The mean concentrations were compared using one-way and two-way repeated-measures analysis of variance (ANOVA) and Tukey’s multiple comparisons tests (CAT: catalase; GPX: Glutathione peroxidase; SOD: superoxide dismutase; MDA: malondialdehyde)

**Fig. 3:**
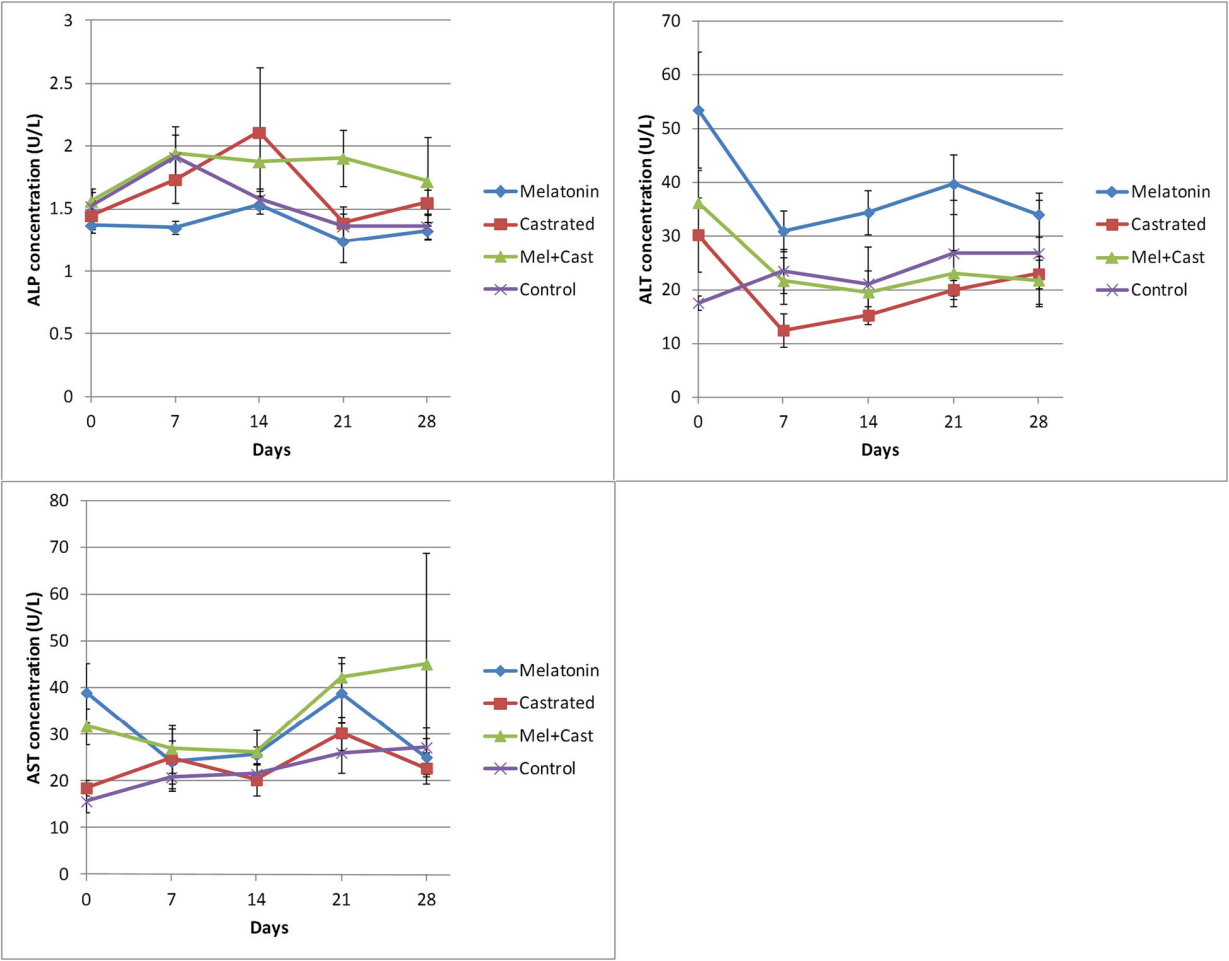
Changes of hepatic enzyme concentrations (Mean ± SEM) in the control, melatonin, castrated, and castrated+melatonin groups during the one-month study. The mean concentrations were compared using one-way and two-way repeated-measures analysis of variance (ANOVA) and Tukey’s multiple comparisons tests (ALP: alkaline phosphatase; ALT: alanine aminotransferase; AST: aspartate aminotransferase)

**Fig. 4:**
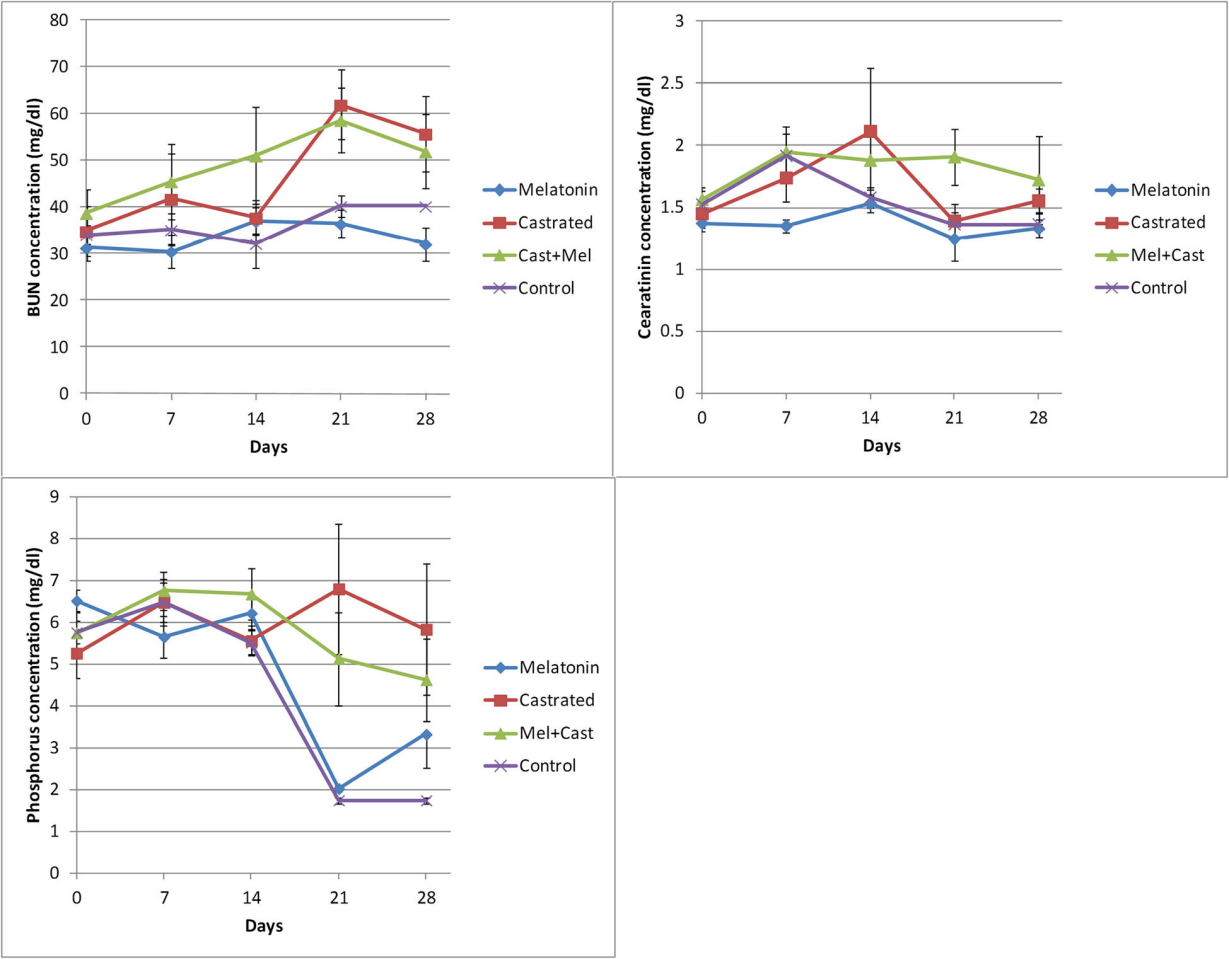
Changes of renal function factor concentrations (Mean ± SEM) in the control, melatonin, castrated and castrated+melatonin groups during the one-month study. The mean concentrations were compared using one-way and two-way repeated-measures analysis of variance (ANOVA) and Tukey’s multiple comparisons tests (BUN: blood urea nitrogen)

**Fig. 5:**
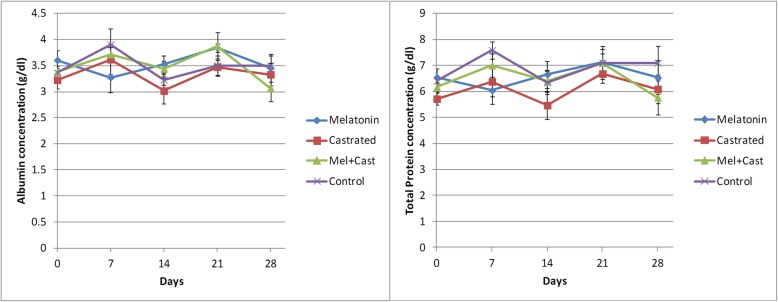
Changes of serum protein (Albumin and total protein) concentrations (Mean ± SEM) in the control, melatonin, castrated and castrated+melatonin groups during the one-month study. The mean concentrations were compared with one-way and two-way repeated-measures analysis of variance (ANOVA) and Tukey’s multiple comparisons tests

The results are summarized and presented in Figs. 2, 3, 4, 5 and Tables 1, 2, 3, 4.

**Table 1:** Mean ± SEM concentrations of antioxidant enzymes and malondialdehyde (MDA) in the melatonin, castrated, control, and castrated and melatonin treated (Cast+Mel) groups during the one-month study. Melatonin was administrated to the melatonin and Cast+Mel groups for 1 month

	CAT (U/gHb)	GPX (U/gHb)	SOD (U/gHb)	MDA (μmol/L)
Control (*n* = 5)	1867 ± 6.30^a^	542.1 ± 16.31^a^	978.2 ± 1.60^a^	8.77 ± 0.01^a^
Melatonin (*n* = 5)	2164 ± 9.83^b^	927.2 ± 13.89^b^	1249 ± 5.58^b^	7.45 ± 0.05^b^
Castrated (*n* = 5)	1710 ± 4.09^c^	368.6 ± 2.68^c^	882.8 ± 2.86^c^	9.72 ± 0.009^c^
Cast+Mel (*n* = 5)	1997 ± 3.29^d^	623.1 ± 1.88^d^	1058 ± 2.11^d^	8.46 ± 0.01^d^

^a,b,c,d^ Different superscript letter in one column indicate significant differences between the groups

Mean concentrations were compared using one-way repeated-measures analysis of variance (ANOVA) and Tukey’s multiple comparisons tests

*CAT* Catalase, *GPX* Glutathione peroxidase, *SOD* Superoxide dismutase, *MDA* Malondialdehyde

**Table 2:** Mean ± SEM hepatic functional enzyme activities in the melatonin, castrated, control, and castrated and melatonin treated (Cast+Mel) groups during the one-month study. Melatonin was administrated to the melatonin and Cast+Mel groups for 1 month

	AST (U/L)	ALP (U/L)	ALT (U/L)
Control (*n* = 5)	22.24 ± 2.38	58.22 ± 4.58^a^	23.26 ± 2.38^abc^
Melatonin (*n* = 5)	28.56 ± 4.59	119.5 ± 5.14^b^	38.20 ± 4.10^a^
Castrated (*n* = 5)	23.15 ± 2.06	154.4 ± 6.61^c^	20.46 ± 3.16^bc^
Cast+Mel (*n* = 5)	34.49 ± 3.91	65.92 ± 2.10^d^	24.50 ± 2.98^bc^

^a,b,c,d^ Different superscript letter in one column indicate significant differences between the groups

Mean concentrations were compared using one-way repeated-measures analysis of variance (ANOVA) and Tukey’s multiple comparisons tests

*ALP* Alkaline phosphatase, *ALT* Alanine aminotransferase, *AST* Aspartate aminotransferase

**Table 3:** Mean ± SEM concentration of renal function factors in the melatonin, castrated, control, and castrated and melatonin treated (Cast+Mel) groups during the one-month study. Melatonin was administrated to the melatonin and Cast+Mel groups for 1 month

	BUN (mg/dl)	Creatinin (mg/dl)	Phosphorus (mg/dl)
Control (*n* = 5)	36.63 ± 1.57^ab^	1.59 ± 0.09^ab^	4.78 ± 1.02
Melatonin (*n* = 5)	32.48 ± 1.90^a^	1.33 ± 0.06^a^	5.14 ± 0.80
Castrated (*n* = 5)	45.75 ± 5.01^ab^	1.64 ± 0.13^ab^	5.99 ± 0.28
Cast+Mel (*n* = 5)	49.24 ± 3.33^b^	1.80 ± 0.07^b^	5.79 ± 0.41

^a,b,c,d^ Different superscript letter in one column indicate significant differences between the groups

Mean concentrations were compared using one-way repeated-measures analysis of variance (ANOVA) and Tukey’s multiple comparisons tests

*BUN* Blood urea nitrogen

**Table 4:** Mean ± SEM concentration of total protein and albumin in the blood serum in the melatonin, castrated, control, and castrated and melatonin treated (Cast+Mel) groups during the one- month study. Melatonin was administrated to the melatonin and Cast+Mel groups for 1 month

	Total protein (g/dl)	Albumin (g/dl)
Control (*n* = 5)	6.94 ± 0.28	3.44 ± 0.12
Melatonin (*n* = 5)	6.29 ± 0.34	3.41 ± 0.16
Castrated (*n* = 5)	6.03 ± 0.22	3.30 ± 0.10
Cast+Mel (*n* = 5)	6.48 ± 0.25	3.49 ± 0.14

^a,b,c,d^ Different superscript letter in one column indicate significant differences between the groups

Mean concentrations were compared using one-way repeated-measures analysis of variance (ANOVA) and Tukey’s multiple comparisons tests

### Antioxidants and oxidant concentrations

#### SOD

Castration significantly decreased the SOD levels by 882.8 ± 2.86 in the castrated dogs in comparison with 978.2 ± 1.60 U/gHb in the control group and (*P* < 0.0001). The melatonin administration significantly increased SOD concentration in the Cast+Mel (1058 ± 2.11 U/gHb) dogs in comparison with the control group, and the maximum concentration was observed in the melatonin group (1249 ± 5.58 U/gHb, Table 1). Significant differences were observed between all the groups in all days of sampling (*P* < 0.04), except that between the control vs. Cast+Mel at day 0 and 14 (*P* < 0.05) (Fig. 2).

#### GPX

The minimum and maximum concentration of GPX was observed in the castrated (368.6 ± 2.68) and melatonin (927.2 ± 13.89) groups, respectively. The oral administration of melatonin in the castrated dogs significantly increased the GPX levels (623.1 ± 1.88) in comparison with those of the control (542.1 ± 16.31) and castrated groups (*P* < 0.0001). Significant differences were observed between the melatonin vs. control, melatonin vs. castrated, and melatonin vs. Cast+Mel (*P* < 0.04) groups at all days of sampling (Table 1). Time and the interaction of time and group factors did not influence GPX concentrations during the study, so there were no significant differences between the days of sampling in each of the groups (Fig. 2).

#### CAT

The concentration of the CAT during the study was 1867 ± 6.30 U/gHb in the control group. CAT levels increased significantly by 2164 ± 9.83 U/gHb in the melatonin group in comparison with those of the control, but when they were castrated, a decrease was observed in its concentration compared to that of the control group (1710 ± 4.09 U/gHb, *P* < 0.0001). In the Cast+Mel group, the CAT levels (1997 ± 3.29 U/gHb) increased significantly compared with those of the castrated and control dogs. Also, at all days of sampling, there was a significant difference between the melatonin vs. control, melatonin vs. castrated, and castrated vs. Cast+Mel groups (*P* < 0.002, Table 1). Time and the interaction of time and group factors did not influence the CAT concentrations during the study, so there were no significant differences between the days of sampling in each of the groups (Fig. 2).

#### MDA

We observed a significant increase in the MDA, as an oxidation stress index, in the castrated dogs compared with that of the control group (9.72 ± 0.009 vs. 8.77 ± 0.01 μmol/L). MDA concentration decreased significantly following the oral administration of melatonin in the melatonin (7.45 ± 0.05) and Cast+Mel (8.46 ± 0.01) groups (*P* < 0.0001, Table 1). There were significant differences between the melatonin vs. control, melatonin vs. castrated, melatonin vs. Cast+Mel, and castrated vs. Cast+Mel (*P* < 0.04) groups at days 0 and 28, between melatonin vs. control, melatonin vs. castrated, melatonin vs. Cast+Mel, castrated vs. control, and castrated vs. Cast+Mel (P < 0.04) groups at days 7 and 14, and between melatonin vs. control, melatonin vs. castrated, and castrated vs. Cast+Mel (*P* < 0.008) groups at days 21 of the study. Time and the interaction of time and group factors did not influence MDA concentrations during the study, so there were no significant differences between the days of sampling in each of the groups (Fig. 2).

### Hepatic function enzymes concentrations

#### ALP

The maximum concentration of ALP was observed in the castrated dogs in comparison with that of the control dogs (154.4 ± 6.61 vs. 58.22 ± 4.58 U/L). ALP concentration significantly increased in the castrated and melatonin groups (119.5 ± 5.14 U/L) in comparison with that of the control and Cast+Mel (65.92 ± 2.10 U/L) groups (*P* < 0.0004, Table 2). Also, significant differences were observed between melatonin vs. control, melatonin vs. Cast+Mel, castrated vs. Cast+Mel, and castrated vs. control groups at days 0, 7, and 14 (*P* < 0.03). There was a significant difference between the melatonin vs. control, castrated vs. Cast+Mel, and castrated vs. control groups at day 21 (P < 0.03), and between melatonin vs. castrated, castrated vs. Cast+Mel, and castrated vs. control groups (*P* < 0.002) groups at day 28. Time and the interaction of time and group factors did not influence the ALP concentrations during the study, so there were no significant differences between the days of sampling in each of the groups (Fig. 3).

#### ALT

ALT concentration (38.2 ± 4.1 U/L) in the melatonin group significantly increased in comparison with that of the other groups (castrated: 20.46 ± 3.16 U/L; control: 23.26 ± 2.38 U/L; Cast+Mel: 24.5 ± 2.98 U/L; *P* < 0.05), and there were not any significant differences in the average concentration of ALT between other groups (Table 2). Significant differences were observed between the melatonin vs. castrated, melatonin vs. control, melatonin vs. Cast+Mel, and control vs. Cast+Mel groups (*P* < 0.02) at day 0 of sampling, between the melatonin vs. castrated (*P* = 0.01) groups at days 7 and 14, and between the melatonin vs. castrated (*P* = 0.008) and melatonin vs. Cast+Mel (*P* = 0.03) groups at day 21. The time factor influenced ALT concentration but the interaction of time and group factors did not affect its level. There was a significant difference between day 0 of sampling and days 7, 14, and 28 (*P* < 0.01) in the melatonin group. Also, there was a significant difference in the ALT level between days 0 vs. 7 of sampling (*P* = 0.03) in the castrated group (Fig. 3).

#### AST

The minimum and maximum concentration of AST was observed in the control (22.24 ± 2.38 U/L) and Cast+Mel (34.49 ± 3.91 U/L) groups, and the difference was significant (*P* = 0.04). The differences were not significant between the other groups (Table 2). There were not any significant differences between the groups on each day of the samplings. Time and the interaction of time and group factors did not influence the AST concentrations during the study, so there were no significant differences between the days of sampling in each of the groups (Fig. 3).

### Renal function factors

#### BUN

The minimum and maximum concentration of BUN was observed in the control group and the castrated dogs that were treated with melatonin (32.48 ± 1.9 vs. 49.24 ± 3.33 mg/dl), and the differences were significant (*P* = 0.01). An increased level of BUN was observed in the castrated vs. control (45.75 ± 5.01 vs. 36.63 ± 1.57 mg/dl) groups, but it was not significant between the other group (Table 3). A significant difference was observed between the control vs. Cast+Mel at day 14 of sampling (*P* = 0.02), and between melatonin vs. castrated, melatonin vs. Cast+Mel (*P* < 0.01), control vs. castrated, and control vs. Cast+Mel (*P* < 0.02) at days 21 and 28. The time factor influenced significantly the BUN levels during the study, but the interaction of time and group factors did not affect its concentration. There was a significant difference between days 0 vs 21, days 0 vs. 28, days 7 vs. 21, and days 14 vs. 21 in the castrated group and between days 0 vs. 21 in the Cast+Mel group (*P* < 0.02, Fig. 4).

#### Creatinin

We observed a similar pattern with regard to the creatinine concentration, such that the minimum and maximum levels of creatinine were observed in the melatonin and Cast+Mel groups (1.33 ± 0.06 vs. 1.80 ± 0.07 mg/dl, *P* = 0.01). Castration was effective in increasing the creatinine concentration (1.64 ± 0.13 vs. 1.59 ± 0.09 mg/dl), but the increase was not significant (Table 3). There was a significant difference in the cearatinin levels between the melatonin vs. Cast+Mel groups at days 7 (*P* = 0.04) and 21 (*P* = 0.02) of sampling. Time factor and the interaction of time and group factors did not influence the cearatinin concentration during the study. There was a significant difference between days 0 vs. 14 (*P* = 0.03) and days 14 vs. 21 (*P* = 0.01) in the castrated group (Fig. 4).

No significant changes were observed in the phosphorus concentrations between the groups (Table 3). The time factor and the interaction of time and group factors influenced significantly the phosphorus levels during the study (*P* < 0.0009, Fig. 4). At day 21 of the sampling, there was a significant difference between the melatonin vs. castrated (*P* < 0.0001) and melatonin vs. Cast+Mel (*P* = 0.01) groups. Significant differences were also observed between the control vs. castrated and control vs. Cast+Mel groups at days 21 and 28 of the sampling (*P* < 0.02). There were significant differences in the melatonin group between days 0 vs. 21, days 0 vs. 28, days 7 vs. 21, days 14 vs. 21, and days 14 vs. 28 of sampling (*P* < 0.03). In addition, there were significant differences in the control group between days 0 vs. 21, days 0 vs. 28, days 7 vs. 21, days 7 vs. 28, days 14 vs. 21, and days 14 vs. 28 of sampling (*P* < 0.002).

#### Protein concentrations in blood serum

The total protein levels did not change significantly during the study (Table 4), but the time factor influenced significantly the total protein concentration (*P* = 0.01). There was a significant difference between the melatonin vs. control groups at day 7 of the sampling (*P* = 0.04), but there was not any significant difference between the days of sampling in each of the groups (Fig. 5).

The difference in the albumin concentration was not significant between the groups (Table 4). The time factor influenced albumin levels during the study (*P* = 0.02), and there was only a significant difference between days 21 vs. 28 of sampling in the Cast+Mel group (*P* = 0.04, Fig. 5).

## Discussion

In this study, castration significantly induced oxidative stress (decreasing antioxidant enzymes and increasing MDA concentration) in the study groups compared to the control group, and these results were in agreement with those of other studies. Numerous studies have evaluated the effects of surgical time, complication, postsurgical pain, and systemic stress parameters on oxidant-antioxidant status following open and laparoscopic ovariectomy and ovariohysterectomy in the bitch [19, 25, 26]. The greater the oxidative stress, the more severe the cellular damage would be during the surgery which may cause poor post-operation outcomes. Therefore, any decrease in oxidative stress may prove to be critical [21]. Lee et al. (2012) indicated that oxidative stress was induced by anesthesia and surgical trauma in both laparoscopic and open ovariectomy in dogs [19].

Treating intact dogs with melatonin significantly increased SOD, GPX, and CAT levels and decreased MDA concentration in the study groups in comparison with those of the control group. The administration of drugs with antioxidative actions or natural antioxidants in pharmacological concentrations has been shown to modify oxidative stress and reduce both morbidity and mortality in various patient populations [21, 27]. As regards this subject, there is an increasing clinical interest to use a natural antioxidant, such as melatonin. The antioxidant effects of melatonin have been reported in humans and laboratory animals in clinical and experimental studies [1, 15, 19, 21, 28, 29]. Other effects of melatonin, such as its anti-inflammatory [23, 30], antioxidant and anti-inflammatory [31], and pro-osteogenic agent [32] effects, have been studied in dogs. A significant increase in plasma lipid peroxide (LPO) level, nitrite and nitrate (NOx) level, and erythrocyte oxidized glutathione/reduced glutathione (GSSG/GSH) ratio has been reported following the removal of the premolars and molars in dogs whereas melatonin treatment at the time of surgery has been shown to restore the normal values of these parameters. Also, melatonin was found to slightly increase erythrocyte glutathione reductase (GR) activity without changing GPX activity [31].

According to our results, melatonin could control oxidative stress, decrease the MDA level, and increase the antioxidant activities in the castrated dogs. As mentioned before, surgical procedures, such as laparoscopic or open ovariectomy and ovariohysterectomy, could induce oxidative stress conditions in dogs. Treating ovariectomized rats with melatonin could significantly reduce the NOx and LPO levels and proinflammatory cytokines in the liver compared with those of the untreated rats [15]. The antioxidant effects of melatonin have been revealed in the short- and long-term and before and after surgery in dogs [19, 31]. Therefore, it seems logical to recommend its administration after spaying to reduce short- and long-term side effects of surgery and depilation of sex hormones.

ALT and ALP levels increased in the melatonin group, but the hepatic enzymes in the Cast+Mel group were similar to those of the control group. Also, ALP increased in the castrated dogs, so it can be concluded that the administration of melatonin in intact dogs and castrated dogs increased the hepatic enzymes. Castration might have some direct and indirect adverse effects on the liver enzymes. This was compensated by the melatonin administration in the Cast+Mel group. In other studies, melatonin has shown to protect liver against chemical and diet-induced hepatic damages and oxidative stress following ovariectomy [11–15]. Castration, as a surgical procedure, induced oxidative stress, inflammation, and long-time adverse effects, which could be reversed by the administration of melatonin [8, 15, 21, 25, 31].

BUN and creatinine levels increased significantly in the Cast+Mel group in comparison with those of the control group. Non-significant and high levels of these factors were observed following castration. These results were not in agreement with those of other studies. In other studies, melatonin could protect the kidney against oxidative stress and carcinogens [16–18, 33]. It is believed that the protective effects of melatonin against the side effects of carcinogens are due to its direct free radical scavenging and indirect antioxidant activities [33]. Significant changes in blood urea nitrogen and serum creatinine concentrations only reveal a decrease of more than 75% of the renal functional mass. Therefore, there is a need for markers that allow early detection and localization of renal damage [34]. Bertieri et al. (2015) analyzed the urine of dogs before and after castration and, except for the protein concentration on dipstick tests, all the results for the biochemical and sediment analysis of the urine samples were within the reference limits for all dogs [35].

The mean urinary protein-to-creatinine ratio (UPCR) for sexually intact male dogs was 0.12 (the range is from 0.10 to 0.32). The mean UPCR for all castrated dogs was 0.08 (the range is from 0.05 to 0.69). There was a significant difference between the mean UPCR before and after castration [35]. Some conflicting findings regarding the protective effects of melatonin could be attributed to the low number of animals in each group, the limited time after castration, the sensitivity of BUN, and creatinine assay for the accurate detection of kidney function mass. One of the limitations of the present study was the study sample size and the number of animals in each group which failed to comply with the minimum number of animals needed to be present in each group for parametric statistical analysis. This might have likely limited the power of the statistical analysis. Secondly, there was a lack of baseline variables prior to the intervention to ensure that there were no baseline differences between the groups. However, there was a control group that allowed the researchers to make a comparison between the groups with regard to the baseline and changes which took place during the study in factors studied.

In conclusion, castration induced oxidative stress in dogs, and melatonin could control it by increasing the antioxidant enzymes (CAT, GPX, and SOD) and decreasing MDA. The administration of melatonin in intact dogs increased ALT and ALP concentrations significantly. BUN and creatinine concentrations increased significantly in the castrated dogs that were treated with melatonin.

## Data Availability

Data and materials are presented in the materials and methods section.

## Abbreviations

ALP: Alkaline phosphatase
ALT: Alanine aminotransferase
AST: Aspartate aminotransferase
BUN: Blood urea nitrogen
CAT: Catalase
GPX: Glutathione peroxidase
GR: Glutathione reductase
GSSG/GSH: Oxidized glutathione/reduced glutathione
LPO: Lipid peroxide
MDA: Malondialdehyde
NOx: Nitrite and nitrate levels
SOD: Superoxide dismutase
UPCR: Urinary protein-to-creatinine ratio
